# Alien Plant Monitoring with Ultralight Airborne Imaging Spectroscopy

**DOI:** 10.1371/journal.pone.0102381

**Published:** 2014-07-10

**Authors:** María Calviño-Cancela, Roi Méndez-Rial, Javier Reguera-Salgado, Julio Martín-Herrero

**Affiliations:** 1 Departament of Ecology and Animal Biology, University of Vigo, Vigo, Galicia, Spain; 2 Departament of Signal Theory and Communications, University of Vigo, Vigo, Galicia, Spain; University of New South Wales, Australia

## Abstract

Effective management of invasive plants requires a precise determination of their distribution. Remote sensing techniques constitute a promising alternative to field surveys and hyperspectral sensors (also known as imaging spectrometers, with a large number of spectral bands and high spectral resolution) are especially suitable when very similar categories are to be distinguished (e.g. plant species). A main priority in the development of this technology is to lower its cost and simplify its use, so that its demonstrated aptitude for many environmental applications can be truly realized. With this aim, we have developed a system for hyperspectral imaging (200 spectral bands in the 380–1000 nm range and *circa* 3 nm spectral resolution) operated on board ultralight aircraft (namely a gyrocopter), which allows a drastic reduction of the running costs and operational complexity of image acquisition, and also increases the spatial resolution of the images (*circa* 5–8 pixels/m^2^ at *circa* 65 km/h and 300 m height). The detection system proved useful for the species tested (*Acacia melanoxylon*, *Oxalis pes-caprae*, and *Carpobrotus* aff. *edulis* and *acinaciformis*), with user’s and producer’s accuracy always exceeding 90%. The detection accuracy reported corresponds to patches down to 0.125 m^2^ (50% of pixels 0.5×0.5 m in size), a very small size for many plant species, making it very effective for initial stages of invasive plant spread. In addition, its low operating costs, similar to those of a 4WD ground vehicle, facilitate frequent image acquisition. Acquired images constitute a permanent record of the status of the study area, with great amount of information that can be analyzed in the future for other purposes, thus greatly facilitating the monitoring of natural areas at detailed spatial and temporal scales for improved management.

## Introduction

Biological invasions are a significant component of global change, being a major cause of biodiversity loss and an important threat to fundamental ecosystem processes such as fire and nitrogen cycling [1]–[4]. Invasive alien species are also an important cause of economic losses due to costs of control and eradication as well as to direct and indirect impacts on ecosystem services and land-based industries, such as agriculture and forestry [5], [6].

In this context, an effective management of invasive species becomes a major challenge for biodiversity conservation. To this end, a precise determination of the distribution of invasive plant species and their patterns of spread is essential to determine the severity of the problem and to guide control and eradication efforts [7], [8]. Detection and mapping of invasive plant species is commonly based on field surveys, which are time consuming and labor intensive [9], [10], and often provide limited information, usually confined to small sampling areas [8]. Remote sensing constitutes a promising alternative to field surveys, as it can provide rapid and comprehensive assessments of large areas, and also systematically across areas, becoming especially useful in remote or not easily accessible locations [11], [12].

Optical remote sensing techniques involve the acquisition and analysis of reflectance spectra of terrain elements (e.g. vegetation, soil, rocks, etc.), which depend on the relative amount of light that is absorbed or reflected at different wavelengths by the various materials. Biochemical and structural properties of these elements determine their reflectance patterns, which are very similar for all higher plants [13]. This makes the distinction of plant species particularly difficult, depending on the capacities of different methodologies. In this regard, optical remote sensing techniques differ mainly in spatial and spectral resolution, spectral range, and the number of spectral bands where reflectance is measured. Distinguishing between plant species, as required for detection of invasive plants, often demands a large number of spectral bands and high spectral resolutions, in order to detect subtle differences in reflectance patterns. For this reason, the low number of spectral bands and the low spectral resolution of aerial photographs and multispectral sensors do not usually suffice for this demanding application [11]. In contrast, hyperspectral sensors (also known as imaging spectrometers) measure light reflectance in many narrow, adjacent spectral bands (often >100 bands) and have been shown to provide good results when subtle differences in reflectance patterns are to be detected, offering great potential for the precise monitoring of invasive species [12] and other environmental management applications [14]. Moreover, when used on airborne platforms instead of satellites, very high spatial resolutions can be achieved (e.g. less than a meter), which are often necessary in heterogeneous terrains where the target species appear in small patches. However, despite its great utility, the use of imaging spectroscopy in environmental management has not been widely established, and the main reason for this is its high cost [11].

Airborne hyperspectral sensors used during the last decades to validate the new technology and its numerous applications (e.g. CASI, AVIRIS, HyMap) are expensive, complex, big and heavy, requiring stable aircraft capable of housing them, usually twin-engine fixed-wing aircraft. Thus, in addition to the high costs of purchase, operation and maintenance of the sensors, high costs are also associated with the aircraft used. These are planes of considerable operational complexity, requiring specialized pilots and commercial airports for takeoff and landing, often based far away from the areas of interest, all contributing to limit their availability and to increase the cost. Compulsory flight permits and detailed in-advance planning of flight routes and dates implies that in case weather conditions are unsuitable for image acquisition when the planned dates arrive, high cancellation costs have to be assumed with no results. In practice, all these requirements and costs greatly limit the usefulness of the technology, despite its technological potential.

Thus, a main priority in the development of this technology is to lower its cost and simplify its use to make it more readily available, so that its demonstrated aptitude for many applications in environmental management and other fields (e.g. precision agriculture) can be truly realized. With this aim, we have developed a system for hyperspectral imaging operated on board ultralight aircraft, which allows a drastic reduction of the running costs and operational complexity of image acquisition, and also increases the spatial resolution of the images because it allows low-altitude low-speed flying, not possible with bigger, heavier aircraft (see Table 1). On the other hand, the use of ultralight platforms impose rigorous constrains in size, weight and power consumption, and increases the problems associated with motor-induced vibrations and flight instability, associated with the low inertia of ultralight aircraft (Table 1).

**Table 1:** Pros and cons of ultralight versus non-ultralight aircraft.

	Ultralight	Non-Ultralight
Flight speeds	Flight speed for image acquisition can go down to 65 km/hwith safety, which translates in higher spatial resolution (holdingconstant all the rest). Cruising speed: 145 km/h.Maximum speed 185 km/h.	Stall speeds of 100–150 km/h translates in higher flight speedsfor safety and lower spatial resolution. Cruising speed:280–450 km/h. Maximum speed 350–450 km/h.
Flight autonomy	Lower (3.4 h at cruising speed).	Higher (7 h at cruising speed).
Maximum Payload	280 kg (subjet to local regulations).	2450 kg.
Fuel consumption	Lower (about 13.5 L/100 km at 145 km/h cruise speed, 95octane unleaded gasoline).	Higher (about 100 L/100 km, kerosene Jet A1).
Purchase costs	Lower (about 60,000 euros).	Higher (about 1.5 million euros).
Logistics	Small hangar (maximum aircraft length: 8.5 m, including theremovable rotor, 1.8 m wide, 2.7 m max. height),270 kg empty weight.	Larger hangar (max. aircraft length: 15.8 m, 19.8 m wide, 5,9 mmax. height), 1800–3200 kg empty weight.
	Shorter runway required (70 m for take-off and 0–30 m for landing,an unpaved track suffices). It can be transported to the flightarea by road in a trailer towed by aconventional car.	Longer runway required (usually 1,000 m long and 15 mwide, properly paved for the weight and powerof the aircraft).
Regulations: aircraftcertifications, pilotlicenses and flightplanning.	Lower requirements (check local rules).	Higher requirements (check local rules).
Flight stability	Lower weight translates into lower inertia and stability,although stability is higher for gyropters than fixed-wingultralight aircraft.	Higher weight translates into higherinertia and stability.

Main advantages and disadvantages of using ultralight and non-ultralight aircraft for hyperspectral image acquisition are shown. The characteristics of ultralight aircrafts are based on gyropters, such as MagniGyro M16, and those of non-ultralight aircraft are based on non-ultralight fixed-wing aircraft, such as the DeHavilland DHC-6 Twin Otter 400 or Cessna 340, models frequently used for imagery acquisition.

In this study, we test the capacities of the ultralight system for invasive plant detection, by determining its accuracy (both producer’s and user’s accuracy) in detecting patches of 0.125 m^2^ in size occupied by invasive species. We used as models a set of invasive species (namely *Acacia melanoxylon*, *Oxalis pes-caprae*, and *Carpobrotus* aff. *edulis* and *acinaciformis*) with contrasting spectral features, sizes and growth patterns, with the objective of making generalizations on the system suitability and its limitations.

## Methods

### Ethics statement

All necessary permits to work in the protected areas where the study was carried out were obtained for the study. Permits were approved by the Atlantic Islands National Park and Ses Salines Natural Park authorities.

### Study area and species

The study was carried out in two different coastal areas in Spain, in the Atlantic and Mediterranean coasts. In the Atlantic coast, we chose the Atlantic Islands National Park (42°22′N, 8°56′W, NW Spain), and nearby areas in the mainland. In the Mediterranean coast we chose the Ses Salines Natural Park of Formentera Island and nearby areas (38°43′N, 1°26′W, Balearic Islands).

We worked with two images in Formentera taken on July 2010, focusing on an area 4.8 km long and *circa* 200 m wide along the NE coast (from the Northern tip of the island, in Ses Salines Natural Park, towards Es Pujols) and another in the SE of the island, in Platja Migjorn, 4.3 km long and *circa* 200 m wide. In Cíes and Ons Islands (Atlantic Islands National Park) we worked with two images taken on March 2011, focusing on an area 4.5 km long and 300 m wide along the eastern part of the northern Cíes island, and an area 5 km long (N–S) and *circa* 600 m wide along the eastern part of Ons island. Also in the Atlantic coast, we worked with an image taken on May 2010 on the northern Portuguese coast (near Vila Praia de Ancora), focusing on an area 2.2 km long (N–S along the coast from the northernmost tip of the Portuguese coast to the south) and *circa* 400 m wide. These study areas were chosen for the presence of the target species.

The model species chosen (*Acacia melanoxylon*, *Oxalis pes-caprae*, and *Carpobrotus* aff. *edulis* and *acinaciformis*) are all listed as invasive species in Spain and other areas [15], [16]. *Acacia melanoxylon* is a tree 10–15 m (up to 45 m) high native from Australia which has spread in Africa, Asia, Europe, South America, the United States and islands in the Indian and the Pacific Ocean. In the study area it usually forms compact thickets, although isolated individuals are also frequent, sometimes of small sizes. *Oxalis pes-caprae* and *Carpobrotus* spp. are herbs native from South Africa. *Oxalis pes-caprae* is a small perennial herb that has spread widely across many parts of the world, including Southern Europe, North Africa, the Middle East, Asia (India, Pakistan, China, Japan), Australia, the United States (mostly California) and western South America. It usually appears in pastures, cultivated fields and along roadsides. It propagates profusely through its underground bulbs forming clonal patches of varied sizes. *Carpobrotus* spp. are succulent and evergreen and have spread in Europe, Northern Africa, the Middle East, the United States (California and Florida) and South America, South Pacific islands, Australia and New Zealand. They appear mainly in coastal dunes, beaches and rock cliffs and have very active vegetative propagation, forming dense clonal patches.

Images were acquired on clear and sunny days, at midday and during the months of highest sun elevation (May-July), when illumination conditions are optimal. For *O. pes-caprae*, however, images were taken in March, when it is at its maximum vegetative development, since this species has a very early phenology. Only for this species images were taken during the flowering season, since it flowers very early (it peaks in December-March) and before flowering (October-November), conditions for image acquisition are poor, due to bad weather and low height of the sun. For the rest of species, images were taken during non-flowering season.

### The hyperspectral sensor and aircraft

**Figure 1:**
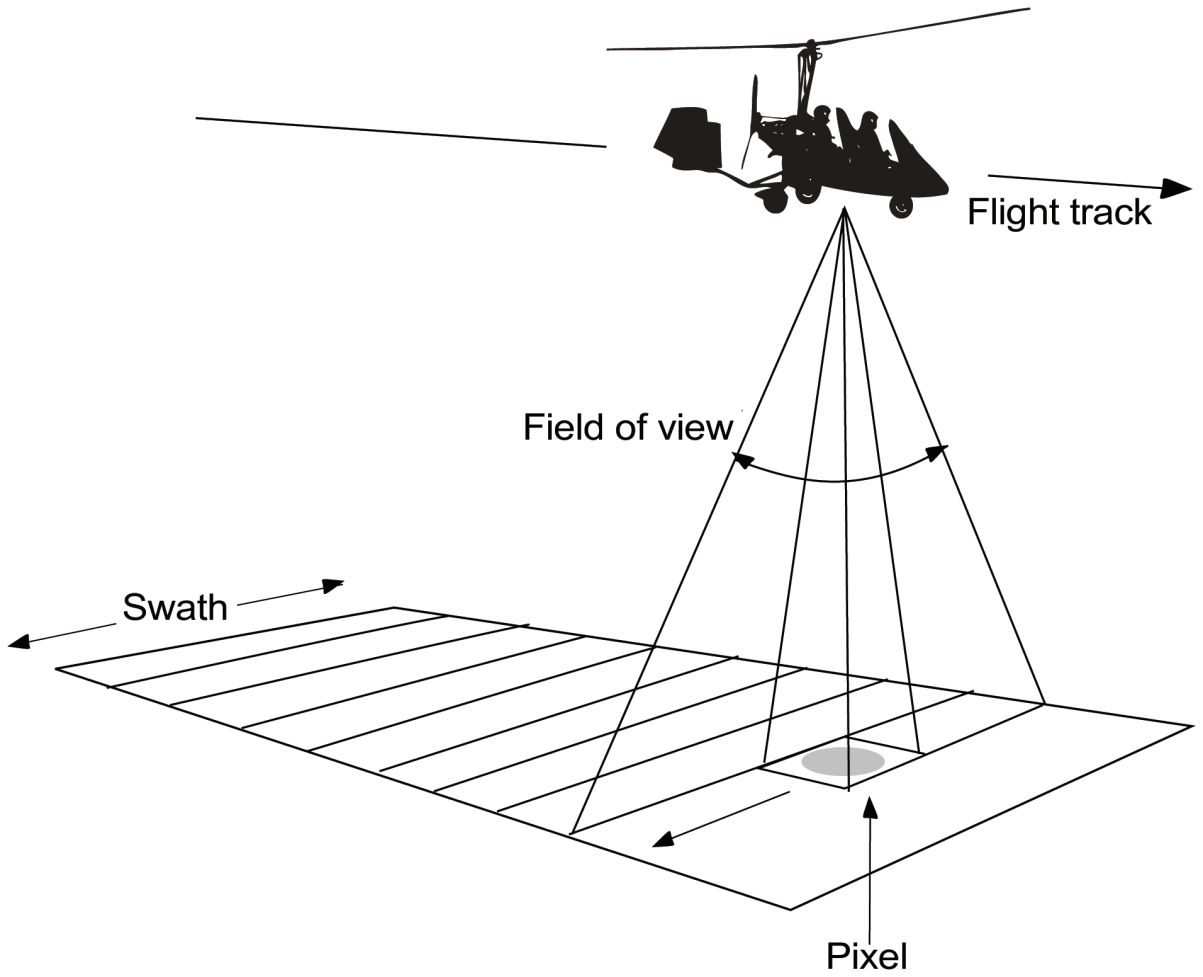
Acquiring images. Image acquisition from a gyropter using a pushbroom sensor.

**Figure 2:**
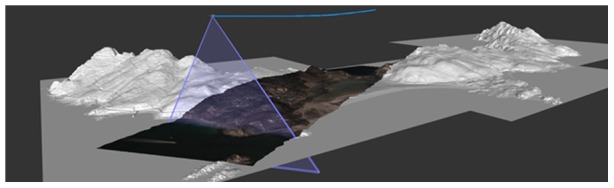
Image projection. Projection of an image taken in the Cíes Islands (Atlantic Islands National Park) on a digital terrain model (DTM).

We used a custom airborne pushbroom hyperspectral sensor integrated in our lab, using custom and off-the-shelf components, with 200 spectral bands in the 380–1000 nm range (visible and near-infrared) with spectral resolution of *circa* 3 nm. Its small size and weight (30 cm×15 cm×15 cm and 4.5 kg) allow us to mount it on ultralight aircraft, namely a gyrocopter (see Table 1). The spatial resolution of the acquired images depends on the forelens of the sensor but also, and very importantly, on the flight height and speed. In pushbroom imagers (Fig. 1 and 2), spatial resolution is defined in two dimensions: along-track (parallel to the flight direction), and across-track (orthogonal to the flight direction). For fixed forelens and focal plane array, flight height determines the across-track pixel resolution while aircraft ground speed together with the sensor integration time determine the along-track pixel resolution [17]. For a given integration time (the time it takes to acquire an image line with all its spectral bands), the along-track resolution decreases with increasing ground speeds. Alternatively, the sensor’s integration time can be shortened, but then fewer photons reach the sensor and signal level decreases [18]. In addition, at a fixed along-track resolution, the amount of information per unit time to be transmitted to the storage subsystem increases with aircraft speed, so that bandwidth, always limited and especially in portable devices, becomes a problem. Ultralight aircraft have the advantage over heavier fixed-wing aircraft of much lower stall speeds, meaning that they can fly at lower speeds without falling. Among ultralight aircraft, gyrocopters (also called autogyros) are well-known for safe flying and maneuverability at low speeds [19]. In addition to an engine-powered propeller to provide forward thrust, similar to that of fixed-wing aircraft, gyrocopters have an unpowered rotor on top that develops lift through autorotation (similar in appearance to a helicopter rotor, but with no engine, so that it is the air flowing through the rotor disc that causes rotation) [19]. This makes gyrocopters especially suitable for our application, since images can be acquired at low speeds (about 60 km/h), compared to conventional hyperspectral sensors such as AVIRIS, CASI or HyMap requiring heavy aircraft that usually loses lift below 120 km/h. Flying our sensor on a gyrocopter, we obtain spatial resolutions of *circa* 5–8 pixels/m^2^ at *circa* 65 km/h and 300 m height (the swath of the image at this height is 220 m).

In addition, gyrocopters are cheaper and simpler to operate than the fixed-wing planes commonly used for hyperspectral image acquisition. No airports are required, just a flat dirt track less than 100 m long. Flight permits with detailed in-advance planning are not needed in most countries, which gives great flexibility, allowing last-minute changes in dates and target areas depending on weather conditions or in situ observations. This avoids the costs of cancelled missions or with useless results (due to low quality images with poor illumination), and greatly increases the availability and therefore the amount of data. Moreover, maintenance costs, fuel consumption and CO_2_ emissions are similar to those of a 4WD, and licenses needed to fly a gyrocopter are less stringent than those needed to fly fixed-wing planes in most countries. On the side of the disadvantages, the small size and low payload of ultralight aircraft impose rigorous constrains in the design of the system regarding size, weight, and power consumption. The effect of motor-induced vibrations has to be neutralized with proper mechanical design and stabilization. In addition, the low weight of this aircraft results in higher flight instability than heavier planes with higher inertia. Gyropters are more stable than other ultralight aircraft but instability is still an issue, especially given the high spatial resolution required. Flight instability results in deformations of the image track that need proper correction for accurate mapping.

### Image pre-processing

#### Geocorrection

Pushbroom image scanners build images by moving along a target track a linear image sensor perpendicular to the flight direction (Fig. 1 and 2). The forward movement of the sensor is usually that of the platform carrying the scanner (plus stabilization artifacts), a gyrocopter in our case. Image lines are acquired and stored, thus progressively building a 2D image. All pushbroom sensors are subjected to flight dynamics, whereby flight trajectories are never ideal, even in the heavier and more stable aircraft. Ultralight aircraft, due to their lower inertia, experience more movements in the three angles of rotation (roll, pitch, yaw) caused by winds or by differences in air density. This results in more deformations of the images, which impose higher demands on the robustness of geocorrection methods [20]. We use specific high-precision and computationally efficient algorithms for geocorrection both to flat earth and to digital terrain models (DTM; obtained with airborne LiDAR) [21].

The process of registering the image lines to a map involves geocorrection (reallocating image pixels to their correct geographic positions) and geocoding (assigning to each pixel an estimation of its correct geographic coordinates). The final result is a georeferenced image or orthoimage.

The parametric approach to geocorrection uses ancillary sensor data, estimating the location and attitude of the camera at the time of acquisition of each line. This is achieved by means of inertial measurement units (IMU) and GPS positioning. These are combined in our system by means of Kalman filtering into a single attitude and heading reference system (AHRS), such as in aircraft autopilot devices, where the information from the GPS and the IMU are combined to cancel out errors from each source. Thus, attitude and position information is available for each image line, such that it is possible to estimate the location of each image pixel on a model of the terrain from the instantaneous position and line of view of the camera at the time of acquisition, within the measurement precision of the AHRS. Residual errors of attitude, position and calibration of the sensor, which cannot be ruled out completely, can be subsequently corrected by means of nonparametric approaches, using salient features of the image and the map, called ground control points (GCPs) [21].

### Ground truthing

In the field, right after the aerial acquisition, with the corrected orthoimages in a rugged sunviewable touch-screen laptop and using a graphic user interface tool, we labeled the different elements of the terrain in areas where the invasive target species was abundant (with patches of different sizes and characteristics) and other representative elements of the terrain were also present (other species, bare ground, buildings, etc.). We used on average a 100 m×100 m area per every 1–2 km long image (200–300 m wide). Patches of different species and other elements of the terrain were easily identified in the images thanks to their high spatial resolution and the presence of distinctive features, avoiding positional errors inherent to GPS positioning, which we used only in uniform areas. We selected training pixels within these labeled areas to train the classifier.

### Classification

Classification of remotely sensed imagery is the process of assigning pixels to discrete categories of terrain elements, i.e. one of the target invasive plant study species or other categories.

We used Support Vector Machines (SVM; [22], [23]) which allow working with high dimensional input spaces [24], thus no band reduction preprocessing of data was required [25]: All 200 bands were used as input vectors. A SVM is a supervised machine learning method that classifies data by a separating hyperplane that provides the best separation between classes in a very high dimensional feature space [26]. The optimal hyperplane is the one that maximizes the distance in feature space between the hyperplane and the nearest positive and negative training example, called the margin. SVMs have high generalization performance and the training can be performed very efficiently, because, in contrast to other machine learning methods, such as neural networks, it is not random and the optimization problem is convex with a global optimum [27].

### Validation

To assess the accuracy of image classifications we compared the classified image with ground validation data and created an error matrix. For each image, we selected *circa* 100–150 random sample pixels classified as with presence of the target invasive species and *circa* 250–300 pixels of the rest of categories (other species or terrain elements). Validation sample points were selected from the whole image, excluding training points. We determined the rates of omissional errors (or false negatives, when pixels with presence of a target invasive plant were not properly classified) and commissional errors (or false positives, when pixels were classified as with presence of a target invasive plant that is nevertheless absent in the ground) which define the producer’s and user’s accuracy, respectively. We also estimated a kappa coefficient, which provides a measure of the difference in agreement between the classified map and ground validated data against an agreement occurring by chance [28].

There is always a detection threshold related to the spatial resolution of the system and, for all species, there will always be individuals, e.g. seedlings or small plantings, that cannot be detected. Therefore, prior to the validation test, we have to determine the smallest area covered by the target species that will be considered as presence of the species. We established this threshold at 50% of pixel size, i.e. 0.125 m^2^ (35×35 cm) for images of 0.25 m^2^ (50×50 cm) spatial resolution.

### Field spectra collection

Field spectra, used to illustrate the high variability within categories and the similarity between categories in Fig. 6, were recorded using a double-channel spectrometer (Ocean Optics USB2000) mounted in a 2 m height post, in order to measure the reflectance spectra of 0.25 m^2^ patches in the field. Both upwelling, ground-reflected radiation, and downwelling, the incidental radiation measured using a cosine corrector, were recorded simultaneously, in order to correct the upwelling with the downwelling for each patch.

## Results

**Figure 3:**
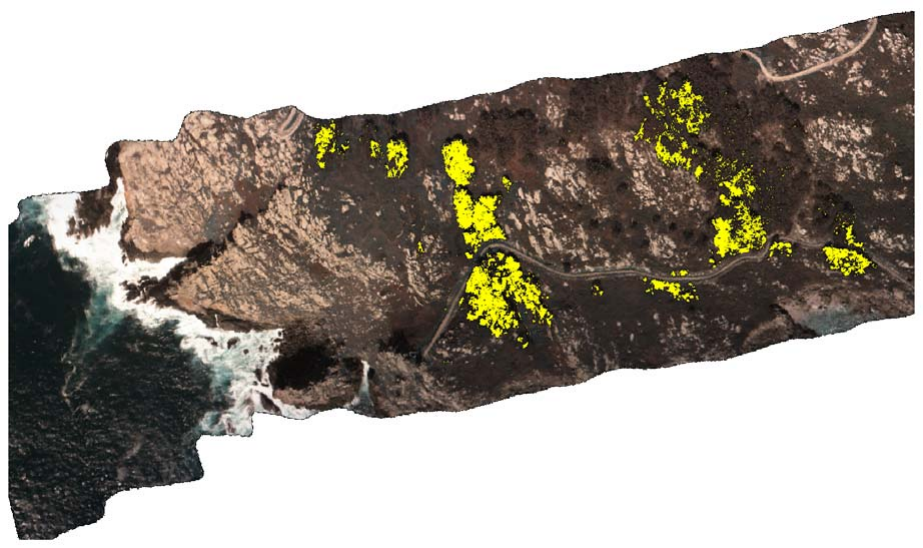
*Acacia melanoxylon* detection. Classified image showing detection of *A. melanoxylon* highlighted in yellow (Cíes Islands, Atlantic Islands National Park, 42°12′50′′N, 8°54′′40′′W). True colour view of a hyperspectral image by the authors.

*Acacia melanoxylon* (Fig. 3) was detected with 100% accuracy when occupying patches bigger than 1 m^2^. For the established threshold of 0.125 m^2^ (50% of pixel size with 0.5 m resolution), we obtained an overall accuracy of 94% (Table 2). We obtained similar results for another species of the same genus, *A. longifolia*, with 97% overall accuracy. In both cases, this accuracy was achieved with non-flowering trees.

**Table 2:** Classification accuracy matrices.

	Ground reference						
Classification	Target species	Others	Row Total	User’s Accuracy (%)	Producer’s Accuracy (%)	Overall Accuracy (%)	Kappa
*Acacia melanoxylon*							
Target spp.	93	7	100	93.0	91.2	95.4	0.888
Others	9	241	250	96.4	97.2		
Column Total	102	248					
*Oxalis pes-caprae*							
Target spp.	100	0	100	100	97.1	99.1	0.979
Others	3	247	250	98.8	100		
Column Total	103	247					
*Carpobrotus* aff. *edulis*							
Target spp.	139	11	150	92.7	97.2	96.7	0.924
Others	4	296	300	98.7	96.4		
Column Total	143	307					

Accuracy matrices with ground reference and classification data for the target species (*Acacia melanoxylon*, *Oxalis pes-caprae* and *Carpobrotus* aff. *edulis*) and other categories.

**Figure 4:**
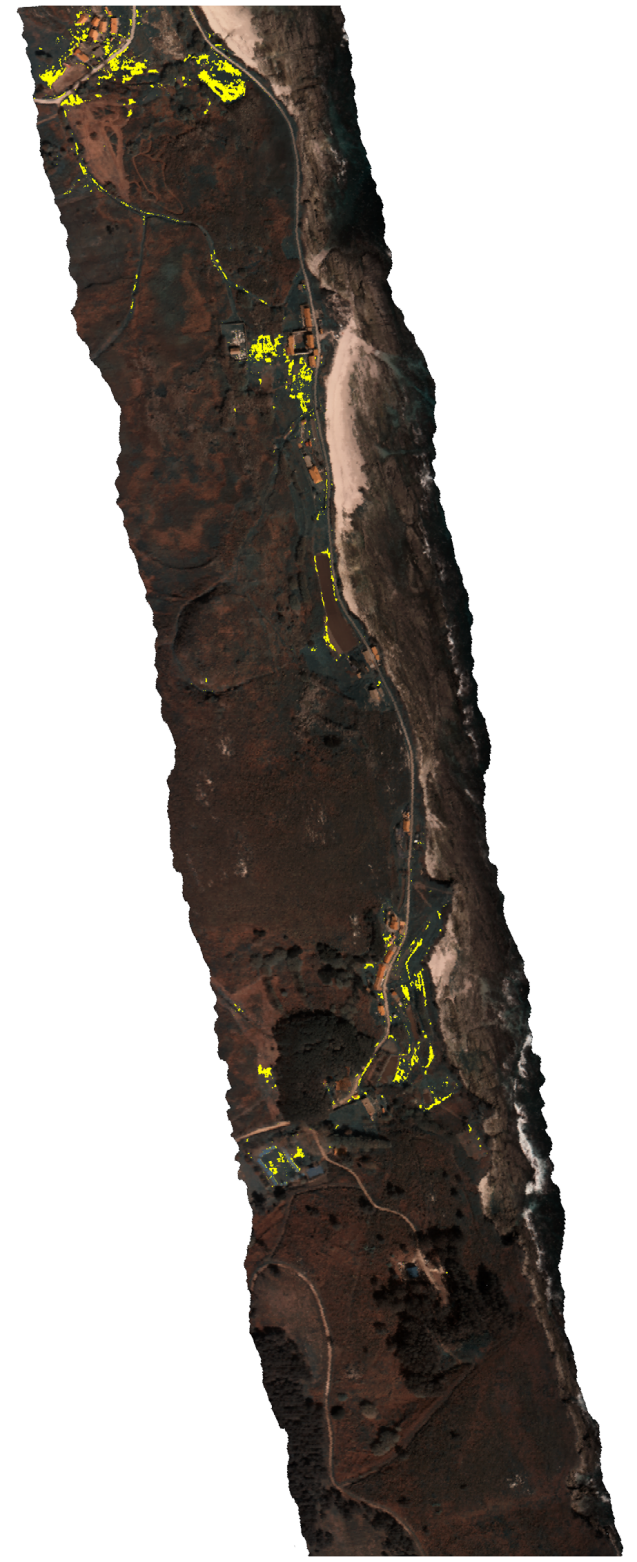
*Oxalis pes-caprae* detection. Classified image showing detection of *O. pes-caprae* highlighted in yellow (Ons Island, Atlantic Islands National Park, 42°22′04′′N, 8°56′06′′W). True colour view of a hyperspectral image by the authors.

*Oxalis pes-caprae* (Fig. 4) overall detection accuracy was 99% for patches *circa* 0.125 m^2^ or bigger (Table 2). There were no false positives (100% user’s accuracy), despite the abundance and simultaneous flowering of gorse (*Ulex europaeus*) in the study area, which also bears yellow flowers.

**Figure 5:**
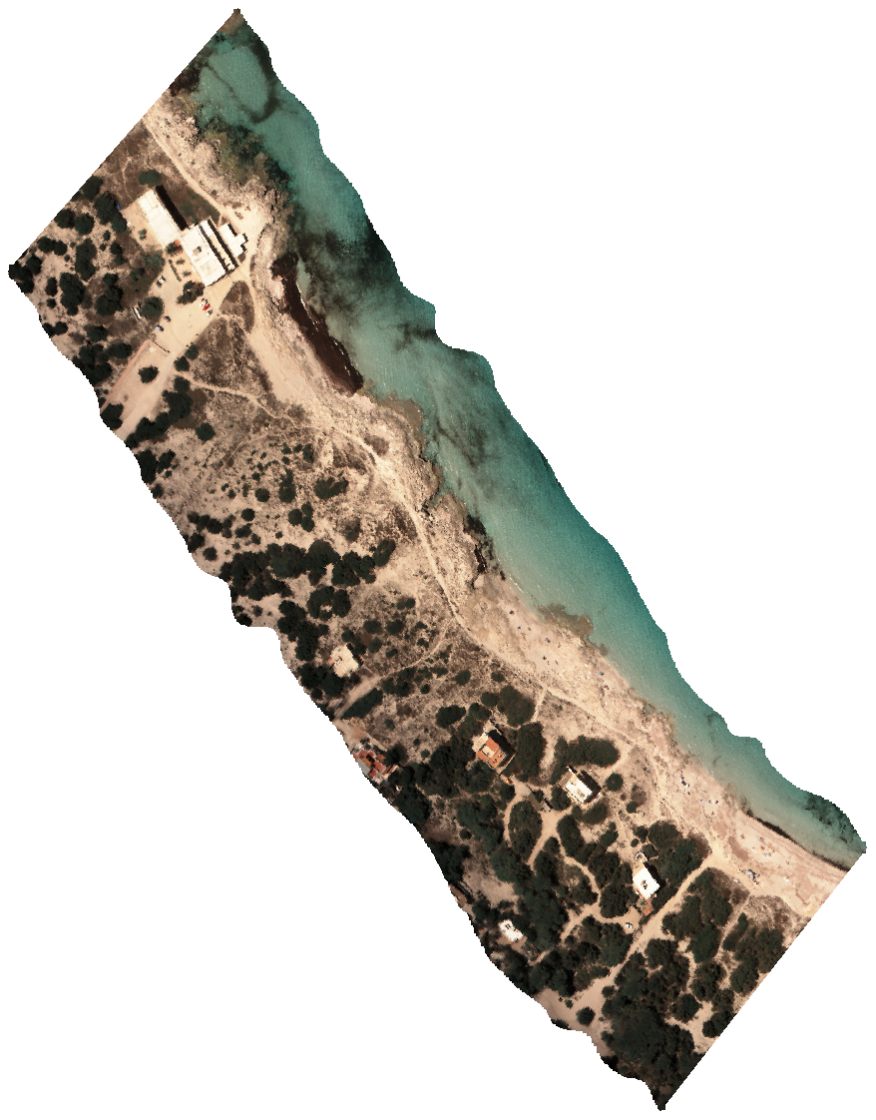
*Carpobrotus* aff. *edulis* detection. Classified image showing detection of *C.* aff. *edulis* highlighted in yellow (Formentera, Balearic Islands, 38°43′52′′N, 1°26′50′′E). True colour view of a hyperspectral image by the authors.

**Figure 6:**
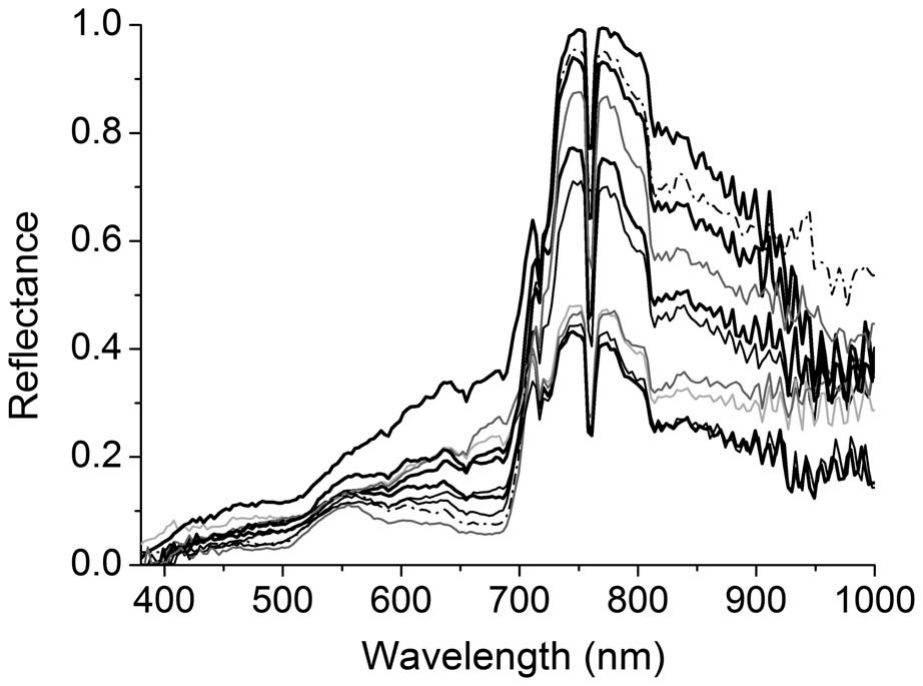
Similarity between *Carpobrotus* aff. *edulis* and accompanying species. Reflectance spectra of *C.* aff. *edulis*; solid black thick line, with healthier and greener patches in the upper lines, going below to patches more affected by water stress) and other species in the same habitat: *Pistacia lentiscus* (dash dotted black line), *Juniperus phoenicea* (solid dark grey line, the upper line corresponds to a tree and the one below to litter, *Pinus halepensis* (solid thin black line, the upper line corresponds with a healthy patch and the one below with a patch of withered needles) and *Crithmum maritimum* (solid light grey line). Notice the variability within categories (e.g. for *C.* aff. *edulis*, *P. halepensis*, and *J. phoenicea*), and the similarity between categories. All spectra were taken at the same dates in an area not bigger than one hectare in Formentera (Balearic Islands).

**Figure 7:**
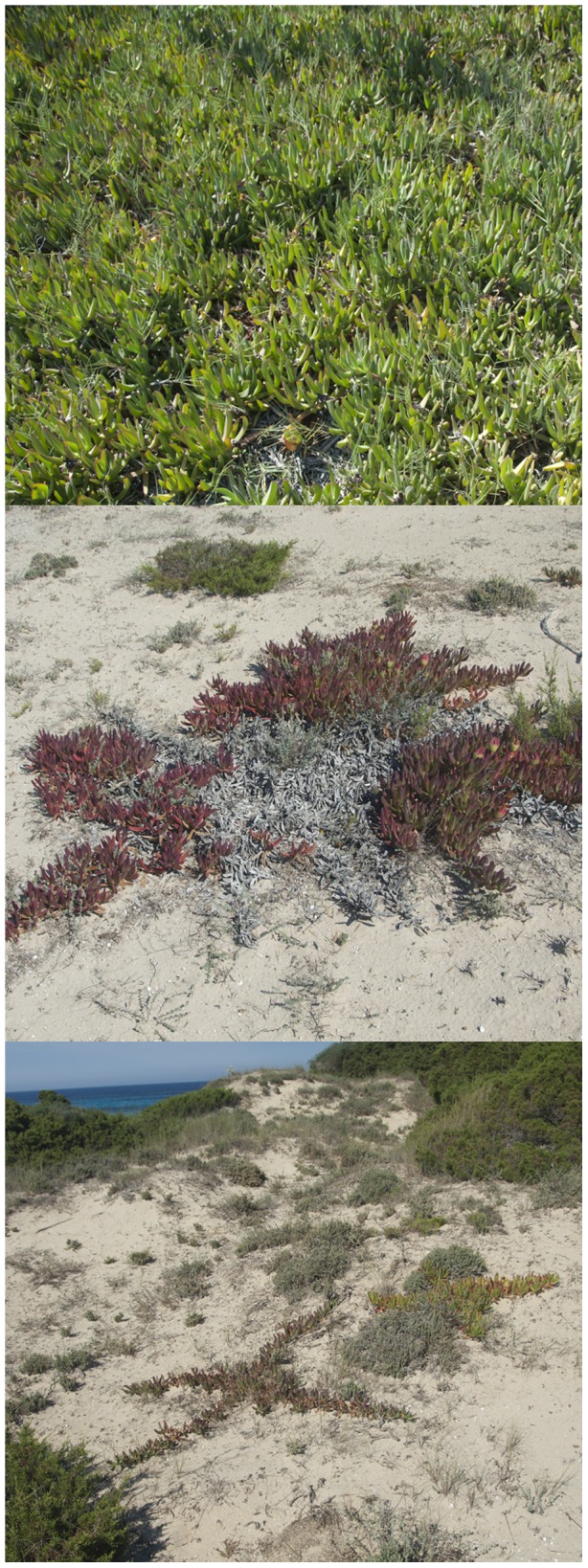
Variability of *Carpobrotus* aff. *edulis* patches. Patches of *C.* aff. *edulis* (Formentera, Balearic Islands) showing the great variability in colour and spatial arrangement: (a) a dense vigorous patch, with high water content, (b) a patch affected by water stress, with dead areas, and (c) linear structures formed by the growth of lateral shoots, which cover small portions of pixels and make detection difficult. Notice the difference between dense and vigorous patches (photo on the upper left), patches with red colour and low water content (upper middle), patches with intermediate colour (bottom left), or lineal structures of different colours that cover small portions of the pixels (upper right, and bottom right and middle).

*Carpobrotus* aff. *edulis* (Fig. 5) constituted a good model for testing the capacities of the detection system, since there was great variability among patches and similarity with other species in the same habitat (Fig. 6). To the human eye, patches varied conspicuously, from vigorous green patches, in shady areas or areas with higher water availability, to red patches, in areas with high insulation and water stress, with varying intermediate tonalities of greenish and oranges (Fig. 7). In addition, due to their growing patterns, the plant can form compact patches that occupy whole pixels rather uniformly, or appear in linear structures that occupy only a small -portion of one or several pixels (Fig. 7). We reached accuracies of 97% (at the established threshold of 0.125 m^2^; Table 2).

Producer’s and user’s accuracies were in general very similar for target species, with differences lower than 3% for *A. melanoxylon*, *A. longifolia*, and *O. pes-caprae* (differences of 0.98%, 1.82% and 2.91%, respectively, with user’s accuracy being higher than producer’s accuracy in all cases; Table 2). For *C.* aff. *edulis*, this difference reached 4.54% and user’s accuracy was lower than producer’s accuracy (92.7% vs. 97.2%). This was mostly due to pixels in patches of *Crithmum maritimum*, a succulent that sometimes shows a reddish color, that were classified as *C.* aff. *edulis*. These were isolated pixels in all cases, so that errors occurred only with small patches occupying only part of the pixel and being mixed with other categories, but not for patches of 0.25 m^2^ or bigger.

## Discussion

The detection system proved useful for the species tested (*A. melanoxylon*, *A. longifolia*, *O. pes-caprae* and *Carpobrotus* aff. *edulis*), with user’s and producer’s accuracy always exceeding 90%. Our results are substantially better than previous studies with the same or similar species [29]–[31], although comparisons are difficult since no reference to the size of patches detected is given in these studies. For instance, Underwood et al. (2003) report user’s and producer’s accuracy of 80% for *C. edulis* (averaging results of scrub and chaparral) when using the highest spectral and spatial resolutions (174 bands, after removing 50 noisy bands, and 4 m; AVIRIS images), and similar results using 30 m spatial resolution, but only *circa* 40% with spectrally degraded images of 6 bands. They concluded that spatial resolution is less important than spectral resolution for detection of invasive species, and attributed their results to the large spatial extent of *C. edulis* infestations in their study area. However, spatial resolution becomes very important with sparse distributions, such as in our study (Figs. 2 to 4). These sparse distributions are typical of early stages of expansion, when control would be most effective and accurate detection more useful [32].

We obtained a good discrimination between classes (even for those with very similar reflectance patterns) in uniform areas where one particular class occupied entire pixels. This is probably due to the large number of bands and high spectral resolution of the images, as highlighted by [29]. Detection errors occurred for small patches of the target or background species, smaller than pixel size, when the target or background species were mixed with other categories in the same pixel, thus generating mixed reflectance spectrums that made discrimination more difficult. Nevertheless, detection accuracy was still high for patches down to 50% of pixel size (0.125 m^2^ for 0.25 m^2^ pixel resolution), which was facilitated by the inclusion of mixed pixels in the training set.

Considering the small size of a newly emerged seedling, there are always individuals that will pass undetected regardless the spatial resolution of the detection technique used. Thus, for every technique there is always a size threshold for detection, and accuracy measures reported in studies always refer, implicitly, to detection of patches that exceed a certain size. However, this size threshold is not explicitly reported in remote sensing studies, which is unfortunate, as it constitutes a necessary reference for comparison between methods, and it is important to judge the utility of a technique for specific purposes. The detection accuracy we report corresponds to patches down to 0.125 m^2^ (50% of pixels 0.5×0.5 m in size), a very small size for many plant species, making it very effective for initial stages of invasive plant spread. This resolution threshold would allow effective control of plants that are not reproductively mature (i.e. do not produce seeds or other reproductive structures) at such small sizes (e.g. most trees and shrubs, and many perennial herbs; [33]). Periodic image acquisition combined with eradication campaigns would ensure that patches that pass undetected due to their small size can be detected in subsequent campaigns, once they exceed the size threshold for detection, but before they are able to reproduce and become a source of invasion. The suitable time intervals between campaigns to assure effective eradication would depend on plant growth rates and size required for reproduction. The detection system would be also effective for species with short-distance dispersal, even if they can reproduce at individual sizes smaller than the detection threshold. In these species, colonization occurs around established foci that are easily detected due to bigger sizes, facilitating the detection of small patches in the field during eradication campaigns. However, the spread of invasive species often occurs through stratified dispersal, with both local spread around established foci and long-distance jumps [34], sometimes facilitated by alternative or non-standard dispersal mechanisms [35], [36]. With long-distance dispersal (e.g. by wind or vertebrate animals), small new patches can be located far away from their parents, making their detection difficult in the field during eradication works, and remote sensing techniques become more necessary for effective control. However, species that can reproduce at sizes smaller than the size threshold for detection (<0.125 m^2^ in our system, e.g. *Arctotheca calendula*) and have effective long-distance dispersal mechanisms are especially problematic for management, since they are hardly detectable by any remote sensing technique and also in field surveys, making population control especially challenging [37].

When resorting to supervised classification methods, ground-truthing is always a critical issue requiring considerable resources, and with a high potential impact on the end result. Based upon our own experience, we advocate ground truthing directly on the geocorrected images themselves, instead of systematically resorting to GPS as a delayed intermediate, much of the time unnecessary, between the terrain and the image. The usual procedure, whereupon GPS coordinates of labeled patches on the terrain are taken in the field and then afterwards labels are assigned to image pixels in the lab by means of the GPS coordinates, involves two sources of error that can be avoided, if not in all, in many cases: the GPS error of the field device and the geocoding error of the image. Instead, we propose going to the field right after the aerial campaign, and to perform the labeling of pixels directly on the images by visual inspection of both, the terrain and the image. Considering high spatial resolution imagery, the terrain is usually heterogeneous with a great variety of salient points that can be used to locate within pixel precision all the necessary features in the image. Only in homogeneous areas this accurate matching of pixels and terrain features can be difficult, but precisely this homogeneity makes less critical the positional accuracy, because in truly homogeneous areas all pixels belong to the same class. Note that we are not advocating labeling only salient features of the image. The procedure for choosing the ground truth plots must be the same as usual: random, ensuring variability, representativity and significance. It is the way of locating, and therefore labeling, the pixels in the image that we propose to change, increasing the accuracy of the labeling and decreasing the labour. This is now possible due to the evolution of portable devices with enhanced graphic and storage capabilities, in connection with adequate, purposedly-designed labeling software.

The use of a gyrocopter as sensor platform allows image acquisition at high spatial resolutions, which enhances the detection ability of the system. In addition, its low operating costs, similar to those of a 4WD ground vehicle, facilitate frequent image acquisition, which adds in monitoring of invasion patterns, allowing a detailed account of temporal and spatial patterns of colonization and establishment. It also facilitates eradication efforts for species with no reproductive capacity at sizes smaller than the detection threshold, as previously commented. Moreover, although images are acquired with a specific goal (e.g. the mapping of invasive species), they constitute a permanent record of the status of the study area, with great amount of information that can be analyzed in the future for other purposes. Periodic acquisition of hyperspectral images can thus greatly facilitate the monitoring of natural areas at detailed spatial and temporal scales for improved management. However, this can only be realistically considered with methods providing good quality images at low costs, such as the system here described. We are currently working in further miniaturization of the system for its operation from small unmanned aerial vehicles (UAV) [38]. However, even if payload has greatly improved in the last few years, making possible the installation of between 3 and 5 kg of imaging and navigation equipment on devices *circa* 1 m of wingspan, flight time of small UAVs, mostly depending on the energy density capability of batteries, is still too low as to consider small UAVs practical for repeated mapping of moderately large areas. This is expected to change in the near future with further developments in battery technology driven by mainstream consumer goods such as portable electronic devices and electric cars. In the meantime, ultralight aircraft, and especially gyrocopters, seem to be the most practical tool for the task.
