# Correction: Alien Plant Monitoring with Ultralight Airborne Imaging Spectroscopy

**DOI:** 10.1371/journal.pone.0112031

**Published:** 2014-10-22

**Authors:** The PLOS ONE Staff

There is an error in Figure 5, “*Carpobrotus* aff. *edulis* detection.” Please see the corrected Figure 5 here.

**Figure 5:**
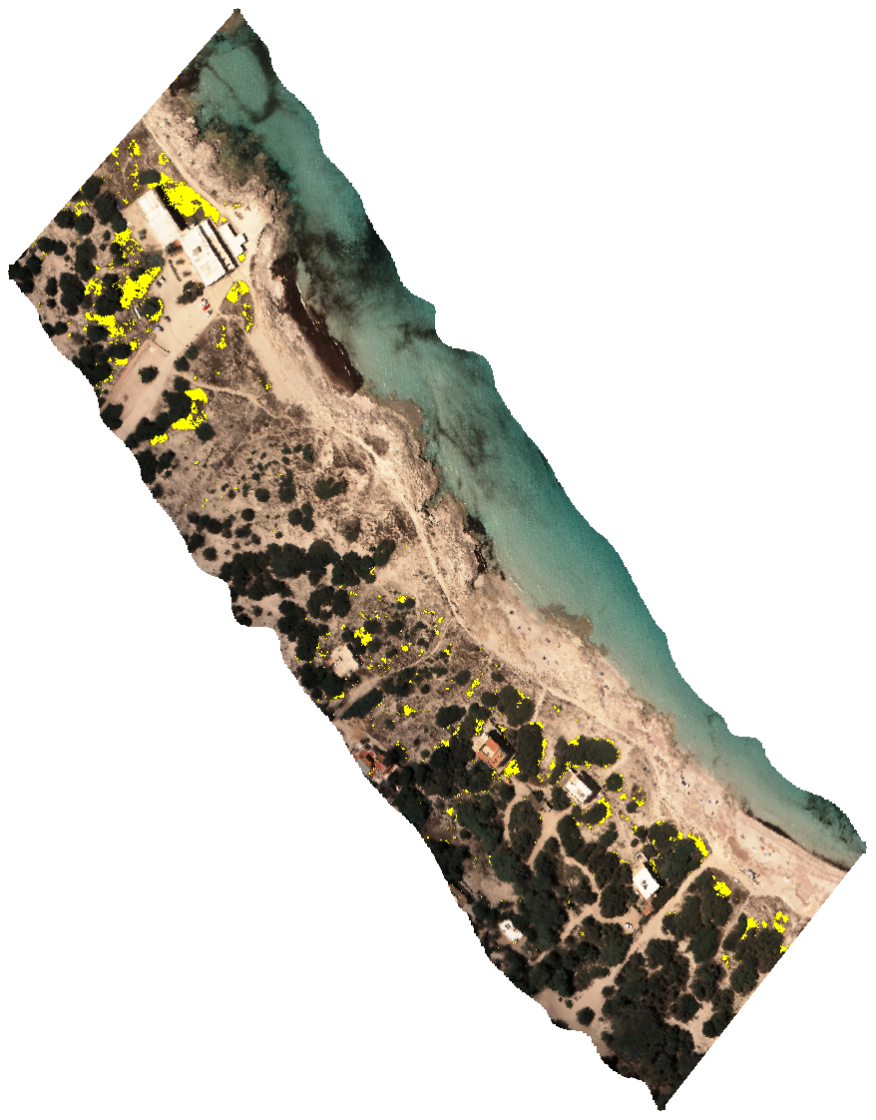
*Carpobrotus* aff. *edulis* detection. Classified image showing detection of *C.* aff. *edulis* highlighted in yellow (Formentera, Balearic Islands, 38°43′52′′N, 1°26′50′′E). True colour view of a hyperspectral image by the authors.
